# Methodological management of end-of-life decision data in intensive care studies: A systematic review of 178 randomized control trials published in seven major journals

**DOI:** 10.1371/journal.pone.0217134

**Published:** 2019-05-28

**Authors:** Sébastien Kerever, Alice Jacquens, Violaine Smail-Faugeron, Etienne Gayat, Matthieu Resche-Rigon

**Affiliations:** 1 Department of Anesthesiology and Critical Care, Lariboisière University Hospital, AP-HP, Paris, France; 2 ECSTRA Team, CRESS, Epidemiology and Statistics Center, Sorbonne Paris Cité, UMR 1153, INSERM, Paris, France; 3 University Denis Diderot—Paris VII, Paris, France; 4 Department of Neuro-Anesthesiology and Intensive Care, Pitié-Salpêtrière University Hospital, Assistance-publique—hôpitaux de Paris, Paris, France; 5 Sorbonne Université, UPMC Paris 6 University, Paris, France; 6 Service d'Odontologie, Hôpital Bretonneau, AP-HP, Paris, France; 7 Unité de Recherches Biomatériaux Innovants et Interface EA4462, Montrouge, France; 8 Université Paris Descartes–Sorbonne Paris Cité, Faculté de Chirurgie Dentaire, Paris, France; 9 Biomarkers in CArdio-Neuro-VAScular diseases (BioCANVAS), UMR-S 942, Inserm, Paris, France; 10 Biostatistics and Medical Information Departments, Saint Louis University Hospital, AP-HP, Paris, France; Universitat d'Alacante, SPAIN

## Abstract

**Background:**

End-of-life (EOL) decisions are a serious ethical dilemma and are frequently carried out in intensive care units (ICUs). The aim of this systematic review was to investigated the different approaches used in ICUs and reported in randomized controlled trials (RCTs) to address EOL decisions and compare the impact of these different strategies regarding potential bias and mortality estimates.

**Methods:**

We identified relevant RCTs published in the past 15 years via PubMed, EMBASE, and CINAHL. In addition, we searched The Cochrane Library and checked registries, including ClinicalTrials.gov to assess concordance between declared and published outcomes. Among the journals we screened were the 3 ICU specialty journals and the four general medicine journals with the highest impact factor. Only RCTs were selected in which in-ICU mortality was the primary or secondary outcome. The primary outcome was information regarding EOL decisions, and the secondary outcome was how EOL decisions were treated in the study analysis.

**Results:**

A total of 178 relevant trials were identified. The details regarding the methodological aspects resulting from EOL decisions were reported in only 62 articles (35%). The manner in which EOL decisions were considered in the study analysis was very heterogeneous, often leading to a high risk of bias.

**Conclusion:**

There is a heterogeneity regarding the management of data on EOL decisions in randomized control trials with mortality endpoints. Recommendations or rules are required regarding the inclusion of patients with potential EOL decisions in RCT analyses and how to manage such decisions from a methodological point of view.

**Trial registration:**

PROSPERO website (CRD42013005724).

## Introduction

End-of-life (EOL) decisions are a serious ethical dilemma in certain medical specialties, particularly for intensivists in the intensive care unit (ICU). Questions regarding the ethical dilemma of EOL decisions have been discussed in an increasing number of scientific articles over the past 10–20 years [1–3]. EOL decisions are typically classified into four categories: (i) do-not-resuscitate (DNR) orders; (ii) withholding treatment; (iii) withdrawal of treatment; and (iv) active shortening of the dying process [4]. DNR orders are instructions or decisions not to attempt cardiopulmonary resuscitation (CPR) in case of cardiac arrest during a patient’s hospital stay. Withholding treatment is the decision not to implement or increase a life-sustaining intervention. Withdrawal of treatment is the decision to actively stop a life-sustaining intervention presently being given. Active shortening of the dying process is a circumstance in which someone performs an act with the specific intention of shortening the dying process [4].

The global ICU mortality was estimated at 13.5% in the European ETHICUS study, which included 31,417 patients between 1999 and 2000 from 37 different European ICUs [4]. In the subset of 4248 ICU patients who died before discharge, 3086 (72.6%) were previously the subject of an EOL decision, which means that an EOL decision was made for approximately 10% of patients during their ICU stay, this type of decision can increase to 66% in other studies like for trauma patient [5]. There was a 68% probability of death within 72 h in the ICU following a decision to withhold life-sustaining treatment and this proportion increased to 93% after decisions to withdraw treatment [4]. In 1997, the LATAREA study, conducted in 113 French ICUs, reported a 53% rate of death in the ICU preceded by a decision to withhold or withdraw therapy [6]. Other studies show that this proportion of patients dying in the ICU with previous limitations increased from 53 to 89% between 2012 and 2016 [2].

Thus, the decision to withhold or withdraw treatment and other EOL decisions may greatly influence the immediate mortality rate and, therefore, could potentially modify the results of ICU studies, especially randomized controlled trials (RCTs), by over- or under-estimating mortality depending on how EOL decisions are considered in the study and/or in the analysis [7,8]. To the best of our knowledge, there is a lack of methodological guidance on how to correctly describe and manage data affected by EOL decisions. Options in the literature range from considering death after EOL decisions as all-cause mortality [9,10] to excluding patients involved in EOL decisions from the analysis [11,12], a clear violation of the intent-to-treat (ITT) principle.

The aims of this systematic review were: (i) to evaluate how many publications of RCTs conducted in ICUs are concerned with EOL decisions; (ii) to identify the different approaches used in RCTs conducted in ICUs to consider and analyze data generated by EOL decisions; and (iii) to compare the impact of these different strategies.

## Materials and methods

### Literature search strategy

Literature searches were conducted using the following databases: PubMed (including MEDLINE, National Library of Medicine, and PubMed Central), Embase (including MEDLINE and Elsevier databases), CINAHL, Google Scholar, and The Cochrane Library. We conducted a systematic search of RCTs with in-ICU mortality as the primary or secondary outcome. Searches were performed using the term “mortality” as a keyword, specified in three different ways (as a MeSH Term, a Subheading, or an All Field term): "Intensive Care Units"[MeSH] AND ("Mortality"[MeSH] OR "mortality"[Subheading] OR "mortality"[All Field]) AND Randomized Controlled Trial[ptyp]. Filters were used in addition to the electronic MeSH search. We focused on three of the highest impact factor ICU-specific journals ("*American Journal of Respiratory and Critical Care Medicine*"[Journal] OR "*Critical Care Medicine*"[Journal] OR "*Intensive Care Medicine*"[Journal]), 3 of the highest impact factor generalist journals ("*New England Journal of Medicine*"[Journal] OR "*JAMA*: *Journal of the American Medical Association*"[Journal] OR "*The Lancet*"[Journal]), and a journal with a methodological tropism ("*BMJ* (Clinical Research edition)"[Journal]). Because the terms “withholding treatment” and “withdrawing treatment” were introduced in the MeSH thesaurus in 2002, we limited our research to trials published after 2002. The systematic review was last updated in March 2018. The four databases were explored using the same keywords, and no additional publications were identified on this topic or methodological rules for managing EOL decision data. Finally, the Cochrane Library was explored to identify systematic reviews or meta-analyses using the keywords “withholding treatment” or “end-of-life decision” or by exploring the topic “ethics”; however, no publications were identified stating how this type of data was managed.

#### Study eligibility criteria

All the potentially relevant abstracts were retrieved, and one author (SK) evaluated the reporting of the full text of the selected articles using a standardized data extraction form. All the data were extracted by two independent researchers (SK, AJ), and items with a low level of agreement were discussed and, if necessary, the abstract was reviewed by a third investigator (MRR) who made the final decision. We hand searched the reference lists of original studies and extracted relevant articles, of which all the potentially relevant studies identified during the abstract search were retrieved and reviewed. Eligible for inclusion were RCTs evaluating ICU mortality either as a primary or secondary outcome. Patients were eligible for inclusion if they were treated in an emergency department, prehospital ambulance care, or another ward of the hospital; however, they must have been involved in an ICU admission or protocol. Articles reporting only statistical plans, methodological plans, and brief reports were excluded from the review as were studies including only patients concerned with EOL decisions or palliative care. The inter-rate agreement between authors for the study selection was measured using Cohen’s kappa statistics (k = 0,82 [0,74; 0,91], almost perfect or perfect agreement).

#### Data extraction

The main outcome of this systematic review was the presence of patients affected by EOL decisions. The information presented in the full text of the article, including the presence and number of patients affected by EOL decisions, was screened and listed on a chart. We also noted whether EOL data were presented on the flow chart and fully described. In addition, methods used to analyze and manage these data were also noted (if available), specifically how these data were included in the analysis of the primary outcome of the study. Information regarding the possible use of survival analysis methods and possible censoring of patients with EOL decisions was also recorded as well as the time interval considered (time to actual death or time to EOL decision).

We also noted the presence of specific analyses concerning EOL decision data, such as sensitivity analysis, and localization of these data on the flow chart. When available, appendix documents, including supplementary documents and Web appendices, were considered and examined.

We also compared the primary and secondary outcomes of studies specified in the trial registries with those reported in the published articles. Thus, items were recorded to determine whether the study’s framework was initially designed as a mortality study and whether the change in primary outcome was linked to the EOL decision data, which could affect the mortality results of the trial.

The agreement between authors for the data extraction was measured using agreement rate (98,4%, 152 non-identical data for 9476 information collected) and Cohen's kappa coefficient (k = 0,97 95%CI [0,96; 0,98]). Cohen's kappa coefficient or ICC were also calculated for qualitative and quantitative variables respectively and presented in **Table 1** and **Table 2**.

**Table 1:** Characteristics of articles eligible for the systematic review.

**Study characteristics**	**Total** **(n = 178)**	**With EOL decision information** **(n = 62)**	**Without EOL decision information** **(n = 116)**	***p***	***agreement***
**Journal name**					
AJRCCM	12 (7%)	4 (6%)	8 (7%)	*0*.*04*	*k = 0*.*98 [0*.*94; 1]*
BMJ	1 (1%)	1 (2%)	0		
CCM	74 (42%)	21 (33%)	53 (46%)		
ICM	44 (24%)	11 (19%)	33 (28%)		
JAMA	15 (8%)	9 (14%)	6 (5%)		
LANCET	7 (4%)	3 (5%)	4 (4%)		
NEJM	25 (14%)	13 (21%)	12 (10%)		
**Country of publication**					
Africa	1 (1%)	0	1 (1%)	*0*.*2*	*k = 0*.*93 [0*.*88; 0*.*96]*
Asia	7 (6%)	0	7 (10%)		
Australia	12 (11%)	5 (12%)	7 (10%)		
Europe	47 (41%)	14 (35%)	33 (45%)		
Middle East	4 (4%)	1 (2%)	3 (4%)		
North America	8 (7%)	3 (7%)	5 (7%)		
South America	6 (5%)	3 (7%)	3 (4%)		
United States	29 (25%)	14 (37%)	14 (19%)		
**Type of intervention**					
Procedure	85 (47%)	30 (49%)	55 (47%)	*0*.*4*	*k = 0*.*88 [0*.*82; 0*.*94]*
Device	18 (10%)	8 (13%)	10 (9%)		
Treatment	74 (42%)	23 (37%)	51 (44%)		
Other	1 (1%)	1 (1%)	0		
**Type of ICU**					
Adult	160 (90%)	54 (87%)	106 (92%)	*0*.*4*	*k = 0*.*98 [0*.*93; 1]*
Pediatric	17 (10%)	8 (13%)	9 (8%)		
**Type of disease**					
All admitted patients	14 (8%)	4 (6%)	10 (9%)	*0*.*3*	*k = 1*
Burn	1 (1%)	0	1 (1%)		
Cardiac arrest	2 (1%)	1 (2%)	1 (1%)		
Cardiology	9 (5%)	1 (2%)	8 (7%)		
Infection	44 (26%)	22 (36%)	22 (19%)		
Metabolism	25 (15%)	10 (16%)	15 (14%)		
Neurology	12 (7%)	4 (6%)	8 (7%)		
Other	8 (5%)	3 (5%)	5 (5%)		
Respiratory	55 (32%)	16 (25%)	39 (36%)		
Trauma	2 (1%)	1 (2%)	1 (1%)		

ITT, Intent-to-treat; EOL: End of life. Data are expressed as number and percentage, n (%), or * median [1^st^; 3^rd^ quartile]. Cohen's kappa coefficient [95% CI].

**Table 2:** Quality of reporting and type of analyses performed in eligible articles for the systematic review.

**Study characteristics**	**Total** **(n = 178)**	**With EOL decision information** **(n = 62)**	**Without EOL decision information** **(n = 116)**	***p***	***agreement***
**Blinded**	59 (34%)	26 (41%)	33 (30%)	*0*.*1*	*k = 0*.*82 [0*.*72; 0*.*90]*
**Multicenter**	114 (64%)	48 (78%)	66 (57%)	*0*.*008*	*k = 0*.*99 [0*.*92; 1]*
Number of centers*	3 [1; 17]	7 [2; 25]	2 [1; 10]	*0*.*003*	*ICC = 1*
Number of patients*	170 [100; 500]	300 [120; 720]	140 [86; 360]	*0*.*002*	*ICC = 0*.*99 [0*.*95; 1]*
Age of patients (years)*	60 [54; 64]	60 [55; 62]	60 [53; 64]	*0*.*5*	*ICC = 0*.*99 [0*.*95; 1]*
Length of study (months)*	26 [17; 39]	29 [19; 40]	26 [16; 35]	*0*.*2*	*ICC = 0*.*99 [0*.*94; 1]*
Publication delay (months)*	27 [18; 38]	23 [14; 37]	29 [20; 38]	*0*.*1*	*ICC = 1*
**Regression analysis**	53 (30%)	22 (37%)	31 (26%)	*0*.*3*	*k = 0*.*93 [0*.*84; 0*.*97]*
**Type of regression**					
Logistic	23 (43%)	9 (44%)	14 (43%)	*0*.*8*	*k = 0*.*84 [0*.*66; 0*.*94]*
Cox	28 (53%)	12 (55%)	16 (52%)		
Fine & Gray	1 (2%)	0	1 (4%)		
Quality reporting score*	8 [8; 9]	9 [8; 9]	8 [8; 9]	*0*.*0008*	*ICC = 0*.*87 [0*.*8; 0*.*92]*
**ITT analysis**	161 (91%)	59 (95%)	102 (89%)	*0*.*2*	*k = 0*.*91 [0*.*74; 0*.*97]*
**Flow chart**	121 (68%)	52 (83%)	69 (61%)	*0*.*001*	*k = 1*
**Study in clinical trial registry**	98 (57%)	41 (68%)	57 (50%)	*0*.*02*	*k = 0*.*99 [0*.*94; 1]*
**Conformity of primary endpoint**	83 (84%)	36 (90%)	47 (82%)	*0*.*1*	*k = 0*.*81 [0*.*88; 0*.*96]*
**Conformity of secondary endpoint**	83 (84%)	36 (88%)	47 (84%)	*0*.*4*	*k = 0*.*93 [0*.*58; 0*.*94]*

ITT, Intent-to-treat; EOL: End of life. Data are expressed as number and percentage, n (%), or * median [1^st^; 3^rd^ quartile]. Cohen's kappa coefficient [95% CI]; ICC intraclass correlation coefficient [95% CI]

#### Quality and risk of bias assessment

Two investigators (SK and AJ) assessed the risk of bias of the selected trials (n = 62) by using the risk of bias tool of The Cochrane Collaboration (Random sequence generation, Allocation concealment, Blinding of participant, Blinding of outcome assessment, Incomplete outcome data, Selective reporting, and other bias) [13]. Disagreements were discussed with a third author (MRR). The considerations for defining the overall risk of bias for each trial are available in **S1 Fig**.

An assessment of the quality of reporting in the eligible publication was also performed using a specially developed checklist and was based on the items on the CONSORT checklist statement [14]. The checklist consisted of scoring the following 10 items: (i) specific objectives or hypotheses; (ii) description of the trial design and intervention; (iii) setting and location; (iv) blinding; (v) statistical method used to measure mortality; (vi) number of patients in each arm; (vii) ITT analysis; (viii) recruitment period, start and end; (ix) trial registration; and (x) concordance between declared and published primary outcomes. For each item, a given article was scored as described (1 point) or not described (0 points). All the articles eligible for this systematic review were rated on a scale from 0 (poor quality) to 10 (excellent quality).

### Statistics

The data for continuous variables are presented as medians and first and third quartiles and for qualitative variables are presented as frequencies and percentages as estimations of 95% confidence intervals (95% CIs) when needed. Qualitative variables were compared using Fisher exact test, and continuous variables were compared using the Wilcoxon rank sum test as appropriate. All the tests were 2-sided and a p-value < 0.05 was considered statistically significant. R Statistical Software (version 3.4.4) was used to perform the statistical analyses. Cohen's Unweighted Kappa, and ICC (intraclass correlation coefficient) were calculated using “psy” package version 1.1, 95% CI were generated by bootstrap method using “boot” package version 1.3–20. Distribution of proportions of EOL decisions in the studies has been described by estimating the proportion of EOL in each study with their exact 95% Confidence Intervals. Those data were summarized in a forest plot to highlight the proportion EOL in each study compared to the 10% expected [4].

This systematic review of the literature was performed as recommended by the *Cochrane Handbook for Systematic Reviews of Interventions* **[15]**, reported to conform to the PRISMA (Preferred Reporting Items for Systematic Reviews and Meta-Analyses) statement **[16]** and registered on the PROSPERO website (CRD42013005724) **[17]**.

## Results

### Description of studies included in the systematic review (n = 178)

**Fig 1:**
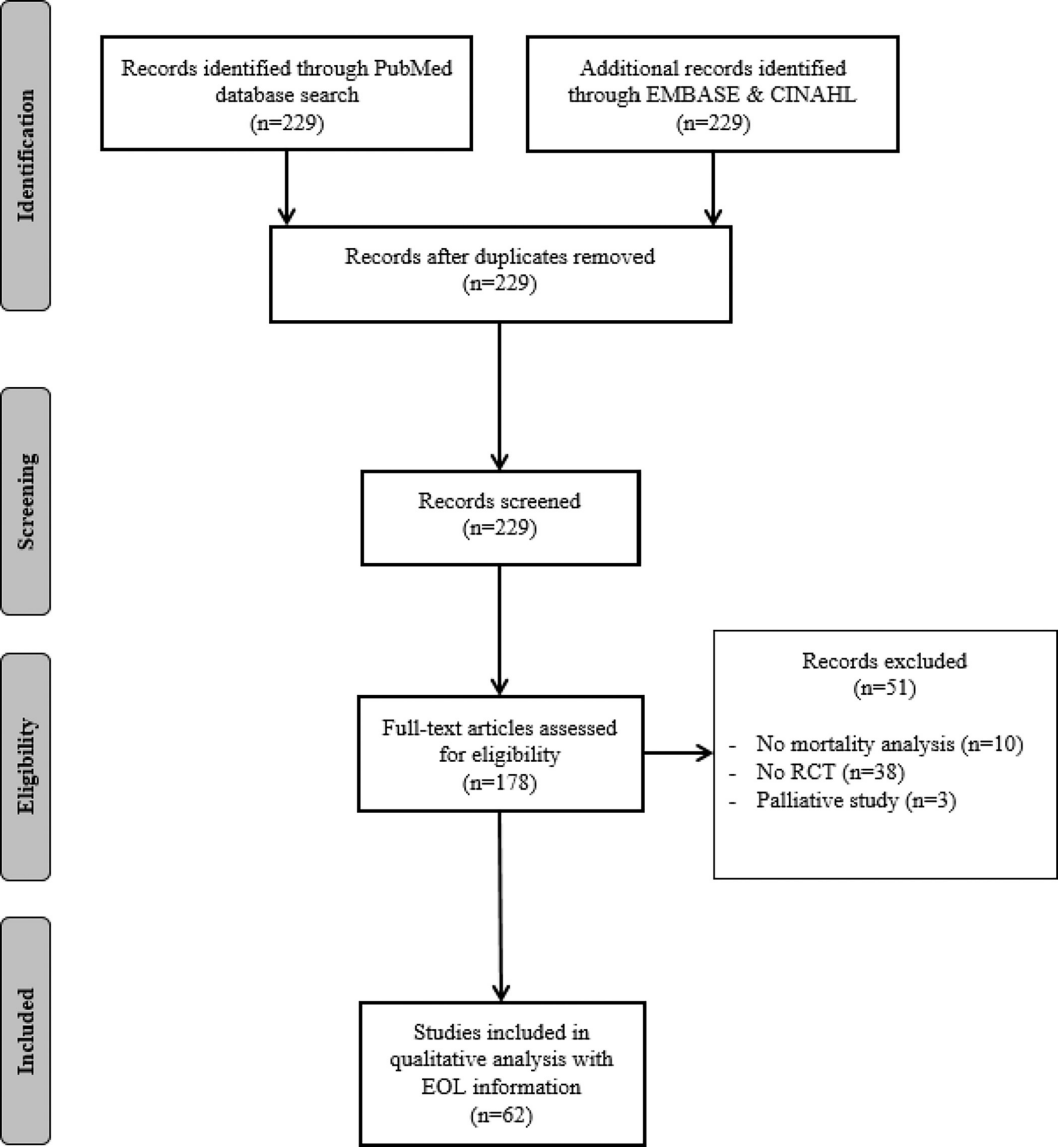
Flow diagram showing the selection procedure for papers.

Overall, 229 articles were identified in the initial literature search. After screening the abstracts, 178 potentially relevant studies were identified and 51 were excluded because they did not meet the inclusion criteria for the systematic review (**Fig 1)**. The PRISMA flow diagram is presented in **Fig 1**. A full-text assessment found that only 35% (n = 62) of the studies reported information on the management of EOL decisions (either in the inclusion criteria or the analyses S1 Text.). **Table 1** show the characteristics of the article included in the systematic review and **Table 2** details the quality of reporting information and type of analyses for all screened articles (n = 178) and for each group.

Most of the 178 RCTs were published in *Critical Care Medicine* (42%) or by European groups (41%). The main types of interventions were procedures (48%) and treatments (42%) and concerned patients admitted for respiratory diseases (32%), infectious pathology (26%), and metabolism disorders (16%). Most of the trials were multicenter (64%) and 34% were blinded. Overall, 91% of the publications reported an analysis using the ITT principle (**Table 2**). The median duration of the studies was 26 months [17; 39], and the median delay between the end of the study and publication was 27 months [18; 38]. The median global score for quality assessment (with 0 being the lowest quality score and ten the highest) for the 178 screened articles was 8 [8; 9].

### Comparison between studies with or without EOL information (n = 62 vs. 116 articles)

Studies providing information regarding EOL decisions were more frequently published in general journals (*New England Journal of Medicine* [*NEJM*], 21% vs. 10%; *Journal of the American Medical Association* [*JAMA*], 15% vs. 5%; *p = 0*.*04*) (**Table 1**). No differences were observed between the studies with or without EOL information in terms of country of publication, type of intervention, or type of ICU. The median number of centers in the two groups of studies was 7 [2; 25] versus 2 [1; 10] (*p = 0*.*003*) with a higher number of centers in the studies providing information about EOL decisions. Overall, 70% of the trials were registered in the group with EOL information and 50% were registered in the group without EOL information, (*p = 0*.*02*). In this systematic review, information on EOL decisions was published more frequently in the highest impact factor journals (*JAMA*, *Lancet*, *NEJM*), and the quality of reporting score was higher in the group of articles reporting information on EOL decisions. The median score for quality of reporting was 9 [8; 9] for those studies reporting information about EOL and 8 [8; 9] for those not reporting information about EOL (*p* = 0.0008).

### Description of the selected studies included in the qualitative analysis (n = 62)

#### Selected studies

Information on EOL decisions was reported in only 62 (35%) of 178 articles (S1 Text), and most of the 62 articles selected for our quantitative analysis were published by investigators from the U.S. (37%) or Europe (34%). In total, 40% of studies were blinded and 77% were multicentric. The median duration of the studies was 29 [19; 40] months, and the delay between the end of the study and the publication date was 23 [14; 37] months. Among the 62 studies included, 57% were registered in an online registry such as ClinicalTrials.gov (i.e. with clearly defined primary and secondary outcomes). Of the registered trials, 83 (86%) had concordance between the registered and published primary and secondary outcomes.

#### Risk of bias

Patients and care providers were blinded in 54 trials (87%), and outcome assessors in 39 trials (63%). Only 38 trials (62%) have a low risk of selective reporting and 51 (82%) low risk of attrition bias. A detailed description of the risk of bias results is provided in **S1 Fig.**

#### End of life decisions

**Fig 2:**
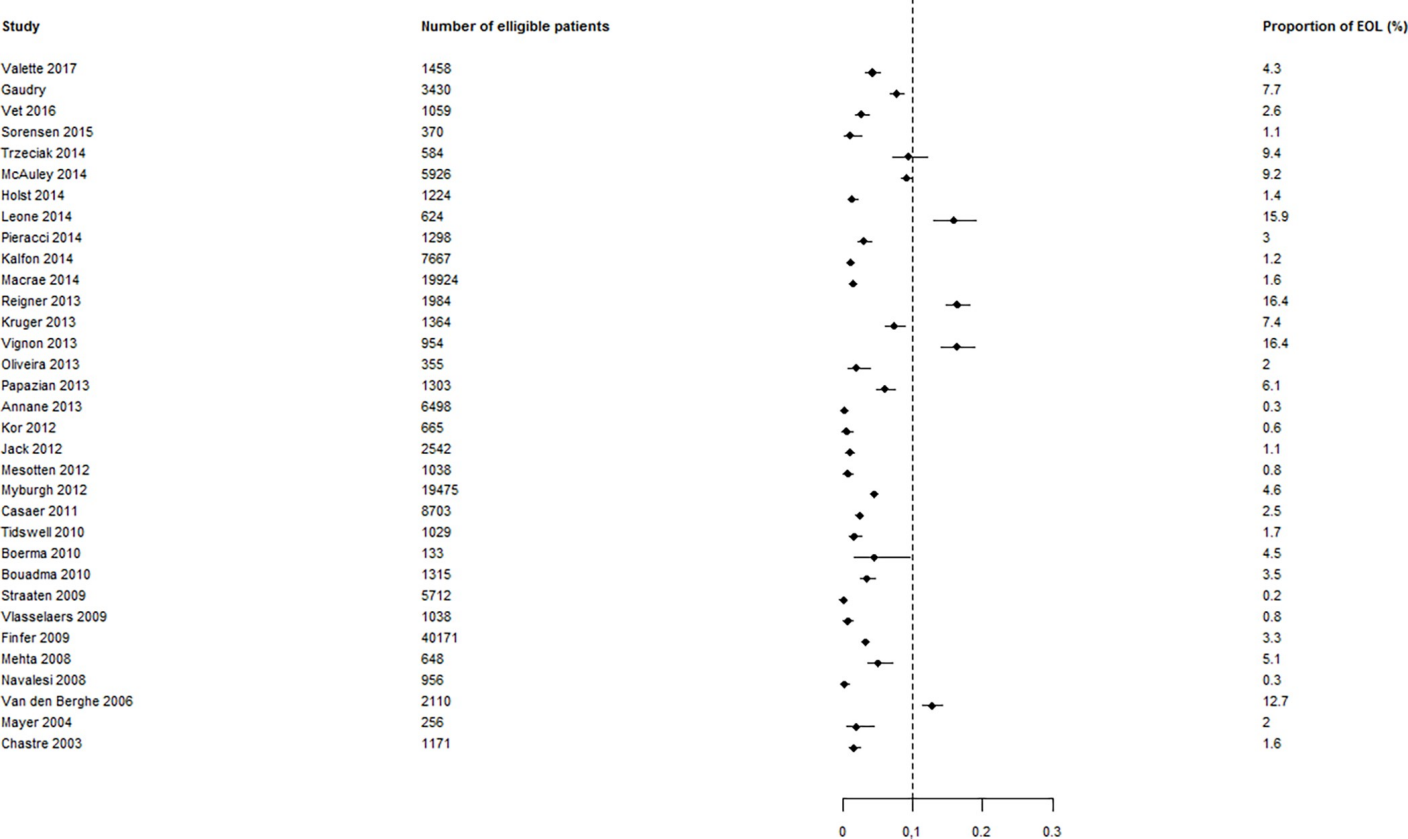
Rate of EOL decisions observed in studies compared to the 10% expected rate (n = 33).

A total of 33 studies reported the overall number of patients affected by EOL decisions; the mean frequency of EOL decisions was 3.56% (95% CI, 3.46%; 3.66%), and the distribution of EOL decision rate was very heterogeneous compared to the 10% rate of expected EOL decisions. **Fig 2** is a graphic representation of this heterogeneity and show the variation of the different rate of EOL decision around the 10% theoretically expected. Only three studies compared patients who were affected or not affected by EOL decisions.

**Table 3** shows information that was available regarding withholding or withdrawing treatment. Three different types of EOL decisions were observed. The most common was withholding treatment (37 articles, 60%), followed by a DNR order (24 articles, 39%) and withdrawing treatment (20 articles, 32%).

**Table 3:** Type of EOL decision and method of managing these data in selected studies.

**EOL decision information**	**(n = 62)**
**EOL decision observed**	39 (63%)
**Rate of EOL decision observed 95% CI**	3.6% [3.5; 3.7]
Type of EOL decision *	
DNR	24 (39%)
Withholding treatment	37 (60%)
Withdrawal of treatment	20 (32%)
Management of EOL decision *	
Non-inclusion criteria	61 (99%)
Exclusion during study	5 (8%)
In overall mortality	2 (3%)

Data are expressed as number and percentage, n (%) or * median [1st; 3rd quartile]

EOL: end of life; DNR: do not resuscitate. Each study could present several characteristics

#### EOL decision management

During this systematic review, we identified three different ways of managing data on patients affected by EOL decisions. The most frequent was to consider EOL decisions as non-inclusion criteria in 61 articles (99%); this technique excludes patients concerned about EOL decisions at the baseline but not during the study itself. The second method, present in five articles (8%), was to consider EOL decisions as exclusion criteria and withdraw patients from the analysis when a new EOL decision was made during the observation or follow-up period [18–22]. The proportion of patients excluded from analysis due to withholding or withdrawing sustaining treatment could affect 90% of the included patients and highlights the fact that EOL as non-inclusion criteria did not prevent the occurrence of EOL during the study or follow-up period [22].

The third method, reported in two articles (3%), was to consider death after an EOL decision as a “classic” way of dying from a disease in the same way as patients who were dying without EOL decisions and to include these data in the overall mortality of the study [23,24]. Only one study specified that during the statistical analysis patients for whom the decision was made to withdraw life support were censored at the time of the decision and not at the time of death [23].

### Use of regression analysis in the selected studies

The statistical analysis of survival/mortality in the 62 studies included regression methods in 22 articles (36%), of which the most common was the Cox proportional hazards model in 12 studies (55%) and logistic regression in 9 (41%). ITT analysis was reported in 59 studies (95%).

## Discussion

The aim of this study was to explore and identify the different ways that withholding or withdrawing life-sustaining treatment are considered in RCT studies conducted specifically in the ICU framework.

In the present study, EOL ranged from DNR to active shortening of the dying process [4]. DNR orders are not always regarded as a restriction or limitation of treatment, but mortality increases after these anticipated directives [25], thus, DNR orders could be considered an intention to withhold supportive care [26]. Active shortening of the dying process, which is synonymous with active euthanasia, is not legal in many countries [27]. We did not observe any declared active shortening of the dying process in the present study.

Our systematic review of 178 RCTs conducted in ICUs found that our primary outcome, information on methods for dealing with EOL decisions, was only declared in 35% of studies despite a global high quality in reporting and global low risk of bias assessment (**S1 Fig).** This illustrates the paradox between a vast amount of literature on ethics, legislation, and medical practice in different countries and the fact that the presence of a potential EOL decision is not considered a methodological issue, notably due to a lack of guidelines on methodology to manage these kind of data, resulting in an increased risk of bias, particularly in cases of post hoc exclusion.

Information on EOL decisions is identified as a marker of high-quality reporting in the CONSORT Statement to highlight the importance of describing the trial population and improving the generalizability of the study’s results: *“4a*. *A comprehensive description of the eligibility criteria used to select the trial participants is needed to help readers interpret the study*. *In particular*, *a clear understanding of these criteria is one of several elements required to judge to whom the results of a trial apply—that is*, *the trial’s generalisability (applicability) and relevance to clinical or public health practice*.*”* [14].

The first and most common method was to consider EOL decisions as non-inclusion criteria (99% of the 61 articles); however, this method of data management leads to questions regarding how representative the selected population is since not including approximately half of the patients dying in ICUs in some pathologies could potentially diminish the relevance of the results [7]. The second point is that not including patients affected by EOL decisions does not avoid the potential need for EOL decisions during the study or follow-up period. The second method of management of EOL decisions, reported in 8% of the 62 analyzed articles, was to exclude patients affected by these decisions from the study or statistical analysis. This method has a high risk of bias, particularly attrition bias, and the ITT analysis in these studies should be reassessed. The third method was to consider a patient dying after an EOL decision like any other patient who dying in the ICU but not following an EOL decision. Such an approach of including these patients in global mortality could bias ICU mortality. Indeed, if mortality is defined at a given time point (such as 28 days) using a logistic model, a patient with an EOL decision who is still alive at 28 days contributes to the underestimation of mortality. On the other hand, by withholding or withdrawing treatment one presumably shortens the delay to death, leading to an overestimation of the mortality rate at that time point. In fact, an EOL decision is a continuous process that appears over time, and standard logistic models are not able to take into account such time-dependent covariates. Since EOL is not known at the baseline, we should avoid such a model if one wishes to consider EOL information in the analysis. Following Resche-Rigon et al, one should probably consider a competing risks framework in which an ICU death without an EOL decision is the outcome of interest and EOL or discharge alive are competing events [28], then the usual approach of competing risks such as Cox or Fine and Gray regression models could be applied. Of course, this issue is particularly true when the number of patients for whom an EOL decision has been made increases.

This systematic review of the literature regarding information about EOL decisions establishes that it is not always easy to obtain instruction about the management of such data. Only 62 of the articles reported details about EOL decisions. Beyond the observed heterogeneous way of analyzing these data, we should insist on improved measures for understanding the weight of EOL decisions in a given study. Indeed, it is important to know when and how these data appear and how they are taken into account. An obvious solution is to visualize the data on EOL decisions in a different part of the flow chart when present or removed from a study, and we noticed that this same flow chart was most frequently available in those articles with information about EOL (83% vs. 61%, p = 0.001). Finally, the management of EOL data in RCTs remains controversial. In most RCTs, the statistical analysis is based on ITT and should include potential EOL decisions. Thus, EOL decisions should be described in RCTs (frequency, time of procedure, precision). Moreover, a precise definition of outcomes such as death without or with an EOL decision should be given as well as the date of the event (considering either the actual date of death or the date of the EOL decision).

Our study has several limitations. First, we restricted our research to seven major journals due to the vast bibliography available in databases such as PubMed and because we focused on ICU survival studies. The choice of journal restriction is also justified by the fact that this systematic review was a primary approach to making a census and identifying the different methods used to analyze data involving EOL decisions. The second limitation is the choice to focus on a specific medical discipline such as the ICU, which is the department with the highest rate of EOL decisions. Similar studies could be conducted in an extended bibliographic search and in other medical disciplines involving EOL decisions such as neurology or oncology.

### Conclusion

Our findings reflect a lack of precision in the EOL description in reports of ICU clinical trials and the heterogeneity of their methodological management. Our systematic review illustrates the need to elaborate on recommendations regarding inclusion/exclusion of patients with EOL decisions in RCTs and to publish rules on reporting and analyzing the data. At least a systematic and precise report of the rate and type of EOL, the delay between EOL and death, and the statistical approach used to consider EOL should be reported for ICU clinical trials.

## Supporting information

S1 Text List of selected studies included in the qualitative analysis (n = 62). (DOCX)

S2 Text Codebook. (DOCX)

S3 Text PRISMA. (DOC)

S1 Fig Risk of bias assessment graphs. (DOCX)
