# Supplementary material for: Methodological management of end-of-life decision data in intensive care studies: A systematic review of 178 randomized control trials published in seven major journals
Source: PLoS One. 2019 May 28;14(5):e0217134. doi: 10.1371/journal.pone.0217134 (PMC6538318; doi:10.1371/journal.pone.0217134)
Supplement: S1 Text — (DOCX) [file pone.0217134.s001.docx]

## S1 Text List of selected studies included in the qualitative analysis (n=62)

1.

Alhazzani W, Guyatt G, Alshahrani M, Deane AM, Marshall JC, Hall R, et al. Withholding Pantoprazole for Stress Ulcer Prophylaxis in Critically Ill Patients: A Pilot Randomized Clinical Trial and Meta-Analysis. Crit Care Med. juill 2017;45(7):1121‑9.

2.

Angus DC, Linde-Zwirble WT, Clermont G, Ball DE, Basson BR, Ely EW, et al. Cost-effectiveness of drotrecogin alfa (activated) in the treatment of severe sepsis. Crit Care Med. janv 2003;31(1):1‑11.

3.

Annane D, Siami S, Jaber S, Martin C, Elatrous S, Declère AD, et al. Effects of fluid resuscitation with colloids vs crystalloids on mortality in critically ill patients presenting with hypovolemic shock: the CRISTAL randomized trial. JAMA. 6 nov 2013;310(17):1809‑17.

4.

Arabi YM, Aldawood AS, Haddad SH, Al-Dorzi HM, Tamim HM, Jones G, et al. Permissive Underfeeding or Standard Enteral Feeding in Critically Ill Adults. N Engl J Med. 18 juin 2015;372(25):2398‑408.

5.

Bergamin FS, Almeida JP, Landoni G, Galas FRBG, Fukushima JT, Fominskiy E, et al. Liberal Versus Restrictive Transfusion Strategy in Critically Ill Oncologic Patients: The Transfusion Requirements in Critically Ill Oncologic Patients Randomized Controlled Trial. Crit Care Med. mai 2017;45(5):766‑73.

6.

Blot F, Similowski T, Trouillet J-L, Chardon P, Korach J-M, Costa M-A, et al. Early tracheotomy versus prolonged endotracheal intubation in unselected severely ill ICU patients. Intensive Care Med. oct 2008;34(10):1779‑87.

7.

Boerma EC, Koopmans M, Konijn A, Kaiferova K, Bakker AJ, van Roon EN, et al. Effects of nitroglycerin on sublingual microcirculatory blood flow in patients with severe sepsis/septic shock after a strict resuscitation protocol: a double-blind randomized placebo controlled trial. Crit Care Med. janv 2010;38(1):93‑100.

8.

Bouadma L, Luyt C-E, Tubach F, Cracco C, Alvarez A, Schwebel C, et al. Use of procalcitonin to reduce patients’ exposure to antibiotics in intensive care units (PRORATA trial): a multicentre randomised controlled trial. Lancet. 6 févr 2010;375(9713):463‑74.

9.

Canadian Critical Care Trials Group. A randomized trial of diagnostic techniques for ventilator-associated pneumonia. N Engl J Med. 21 déc 2006;355(25):2619‑30.

10.

Casaer MP, Mesotten D, Hermans G, Wouters PJ, Schetz M, Meyfroidt G, et al. Early versus late parenteral nutrition in critically ill adults. N Engl J Med. 11 août 2011;365(6):506‑17.

11.

Chastre J, Wolff M, Fagon J-Y, Chevret S, Thomas F, Wermert D, et al. Comparison of 8 vs 15 days of antibiotic therapy for ventilator-associated pneumonia in adults: a randomized trial. JAMA. 19 nov 2003;290(19):2588‑98.

12.

Curley MAQ, Hibberd PL, Fineman LD, Wypij D, Shih M-C, Thompson JE, et al. Effect of prone positioning on clinical outcomes in children with acute lung injury: a randomized controlled trial. JAMA. 13 juill 2005;294(2):229‑37.

13.

Demoule A, Clavel M, Rolland-Debord C, Perbet S, Terzi N, Kouatchet A, et al. Neurally adjusted ventilatory assist as an alternative to pressure support ventilation in adults: a French multicentre randomized trial. Intensive Care Med. nov 2016;42(11):1723‑32.

14.

Dhainaut J-F, Antonelli M, Wright P, Desachy A, Reignier J, Lavoue S, et al. Extended drotrecogin alfa (activated) treatment in patients with prolonged septic shock. Intensive Care Med. juill 2009;35(7):1187‑95.

15.

Finfer S, Bellomo R, Boyce N, French J, Myburgh J, Norton R, et al. A comparison of albumin and saline for fluid resuscitation in the intensive care unit. N Engl J Med. 27 mai 2004;350(22):2247‑56.

16.

Gaudry S, Hajage D, Schortgen F, Martin-Lefevre L, Pons B, Boulet E, et al. Initiation Strategies for Renal-Replacement Therapy in the Intensive Care Unit. N Engl J Med. 14 juill 2016;375(2):122‑33.

17.

Girard TD, Pandharipande PP, Carson SS, Schmidt GA, Wright PE, Canonico AE, et al. Feasibility, efficacy, and safety of antipsychotics for intensive care unit delirium: the MIND randomized, placebo-controlled trial. Crit Care Med. févr 2010;38(2):428‑37.

18.

Gordon AC, Mason AJ, Perkins GD, Stotz M, Terblanche M, Ashby D, et al. The interaction of vasopressin and corticosteroids in septic shock: a pilot randomized controlled trial. Crit Care Med. juin 2014;42(6):1325‑33.

19.

Guntupalli K, Dean N, Morris PE, Bandi V, Margolis B, Rivers E, et al. A phase 2 randomized, double-blind, placebo-controlled study of the safety and efficacy of talactoferrin in patients with severe sepsis. Crit Care Med. mars 2013;41(3):706‑16.

20.

Hillman K, Chen J, Cretikos M, Bellomo R, Brown D, Doig G, et al. Introduction of the medical emergency team (MET) system: a cluster-randomised controlled trial. Lancet. 18 juin 2005;365(9477):2091‑7.

21.

Holst LB, Haase N, Wetterslev J, Wernerman J, Guttormsen AB, Karlsson S, et al. Lower versus higher hemoglobin threshold for transfusion in septic shock. N Engl J Med. 9 oct 2014;371(15):1381‑91.

22.

Hyttel-Sorensen S, Pellicer A, Alderliesten T, Austin T, van Bel F, Benders M, et al. Cerebral near infrared spectroscopy oximetry in extremely preterm infants: phase II randomised clinical trial. BMJ. 5 janv 2015;350:g7635.

23.

Jack T, Boehne M, Brent BE, Hoy L, Köditz H, Wessel A, et al. In-line filtration reduces severe complications and length of stay on pediatric intensive care unit: a prospective, randomized, controlled trial. Intensive Care Med. juin 2012;38(6):1008‑16.

24.

Jansen TC, van Bommel J, Schoonderbeek FJ, Sleeswijk Visser SJ, van der Klooster JM, Lima AP, et al. Early lactate-guided therapy in intensive care unit patients: a multicenter, open-label, randomized controlled trial. Am J Respir Crit Care Med. 15 sept 2010;182(6):752‑61.

25.

Kalfon P, Giraudeau B, Ichai C, Guerrini A, Brechot N, Cinotti R, et al. Tight computerized versus conventional glucose control in the ICU: a randomized controlled trial. Intensive Care Med. févr 2014;40(2):171‑81.

26.

Kor DJ, Kashyap R, Weiskopf RB, Wilson GA, van Buskirk CM, Winters JL, et al. Fresh red blood cell transfusion and short-term pulmonary, immunologic, and coagulation status: a randomized clinical trial. Am J Respir Crit Care Med. 15 avr 2012;185(8):842‑50.

27.

Kruger P, Bailey M, Bellomo R, Cooper DJ, Harward M, Higgins A, et al. A multicenter randomized trial of atorvastatin therapy in intensive care patients with severe sepsis. Am J Respir Crit Care Med. 1 avr 2013;187(7):743‑50.

28.

Lacherade J-C, De Jonghe B, Guezennec P, Debbat K, Hayon J, Monsel A, et al. Intermittent subglottic secretion drainage and ventilator-associated pneumonia: a multicenter trial. Am J Respir Crit Care Med. 1 oct 2010;182(7):910‑7.

29.

Lellouche F, L’Her E, Abroug F, Deye N, Rodriguez PO, Rabbat A, et al. Impact of the humidification device on intubation rate during noninvasive ventilation with ICU ventilators: results of a multicenter randomized controlled trial. Intensive Care Med. févr 2014;40(2):211‑9.

30.

Leone M, Bechis C, Baumstarck K, Lefrant J-Y, Albanèse J, Jaber S, et al. De-escalation versus continuation of empirical antimicrobial treatment in severe sepsis: a multicenter non-blinded randomized noninferiority trial. Intensive Care Med. oct 2014;40(10):1399‑408.

31.

López A, Lorente JA, Steingrub J, Bakker J, McLuckie A, Willatts S, et al. Multiple-center, randomized, placebo-controlled, double-blind study of the nitric oxide synthase inhibitor 546C88: effect on survival in patients with septic shock. Crit Care Med. janv 2004;32(1):21‑30.

32.

Macrae D, Grieve R, Allen E, Sadique Z, Morris K, Pappachan J, et al. A randomized trial of hyperglycemic control in pediatric intensive care. N Engl J Med. 9 janv 2014;370(2):107‑18.

33.

Mayer SA, Kowalski RG, Presciutti M, Ostapkovich ND, McGann E, Fitzsimmons B-F, et al. Clinical trial of a novel surface cooling system for fever control in neurocritical care patients. Crit Care Med. déc 2004;32(12):2508‑15.

34.

McAuley DF, Laffey JG, O’Kane CM, Perkins GD, Mullan B, Trinder TJ, et al. Simvastatin in the acute respiratory distress syndrome. N Engl J Med. 30 oct 2014;371(18):1695‑703.

35.

Mehta S, Burry L, Martinez-Motta JC, Stewart TE, Hallett D, McDonald E, et al. A randomized trial of daily awakening in critically ill patients managed with a sedation protocol: a pilot trial. Crit Care Med. juill 2008;36(7):2092‑9.

36.

Mesotten D, Gielen M, Sterken C, Claessens K, Hermans G, Vlasselaers D, et al. Neurocognitive development of children 4 years after critical illness and treatment with tight glucose control: a randomized controlled trial. JAMA. 24 oct 2012;308(16):1641‑50.

37.

Myburgh JA, Finfer S, Bellomo R, Billot L, Cass A, Gattas D, et al. Hydroxyethyl starch or saline for fluid resuscitation in intensive care. N Engl J Med. 15 nov 2012;367(20):1901‑11.

38.

National Heart, Lung, and Blood Institute Acute Respiratory Distress Syndrome (ARDS) Clinical Trials Network, Wiedemann HP, Wheeler AP, Bernard GR, Thompson BT, Hayden D, et al. Comparison of two fluid-management strategies in acute lung injury. N Engl J Med. 15 juin 2006;354(24):2564‑75.

39.

Navalesi P, Frigerio P, Moretti MP, Sommariva M, Vesconi S, Baiardi P, et al. Rate of reintubation in mechanically ventilated neurosurgical and neurologic patients: evaluation of a systematic approach to weaning and extubation. Crit Care Med. nov 2008;36(11):2986‑92.

40.

NICE-SUGAR Study Investigators, Finfer S, Chittock DR, Su SY-S, Blair D, Foster D, et al. Intensive versus conventional glucose control in critically ill patients. N Engl J Med. 26 mars 2009;360(13):1283‑97.

41.

Oksanen T, Skrifvars MB, Varpula T, Kuitunen A, Pettilä V, Nurmi J, et al. Strict versus moderate glucose control after resuscitation from ventricular fibrillation. Intensive Care Med. déc 2007;33(12):2093‑100.

42.

Oliveira CF, Botoni FA, Oliveira CRA, Silva CB, Pereira HA, Serufo JC, et al. Procalcitonin versus C-reactive protein for guiding antibiotic therapy in sepsis: a randomized trial. Crit Care Med. oct 2013;41(10):2336‑43.

43.

Oudemans-van Straaten HM, Bosman RJ, Koopmans M, van der Voort PHJ, Wester JPJ, van der Spoel JI, et al. Citrate anticoagulation for continuous venovenous hemofiltration. Crit Care Med. févr 2009;37(2):545‑52.

44.

Pandharipande PP, Pun BT, Herr DL, Maze M, Girard TD, Miller RR, et al. Effect of sedation with dexmedetomidine vs lorazepam on acute brain dysfunction in mechanically ventilated patients: the MENDS randomized controlled trial. JAMA. 12 déc 2007;298(22):2644‑53.

45.

Papazian L, Roch A, Charles P-E, Penot-Ragon C, Perrin G, Roulier P, et al. Effect of statin therapy on mortality in patients with ventilator-associated pneumonia: a randomized clinical trial. JAMA. 23 oct 2013;310(16):1692‑700.

46.

Pieracci FM, Stovall RT, Jaouen B, Rodil M, Cappa A, Burlew CC, et al. A multicenter, randomized clinical trial of IV iron supplementation for anemia of traumatic critical illness*. Crit Care Med. sept 2014;42(9):2048‑57.

47.

Reignier J, Mercier E, Le Gouge A, Boulain T, Desachy A, Bellec F, et al. Effect of not monitoring residual gastric volume on risk of ventilator-associated pneumonia in adults receiving mechanical ventilation and early enteral feeding: a randomized controlled trial. JAMA. 16 janv 2013;309(3):249‑56.

48.

Rice TW, Mogan S, Hays MA, Bernard GR, Jensen GL, Wheeler AP. Randomized trial of initial trophic versus full-energy enteral nutrition in mechanically ventilated patients with acute respiratory failure. Crit Care Med. mai 2011;39(5):967‑74.

49.

Rice TW, Wheeler AP, Morris PE, Paz HL, Russell JA, Edens TR, et al. Safety and efficacy of affinity-purified, anti-tumor necrosis factor-alpha, ovine fab for injection (CytoFab) in severe sepsis. Crit Care Med. sept 2006;34(9):2271‑81.

50.

Rice TW, Wheeler AP, Thompson BT, deBoisblanc BP, Steingrub J, Rock P, et al. Enteral omega-3 fatty acid, gamma-linolenic acid, and antioxidant supplementation in acute lung injury. JAMA. 12 oct 2011;306(14):1574‑81.

51.

Richard C, Warszawski J, Anguel N, Deye N, Combes A, Barnoud D, et al. Early use of the pulmonary artery catheter and outcomes in patients with shock and acute respiratory distress syndrome: a randomized controlled trial. JAMA. 26 nov 2003;290(20):2713‑20.

52.

Rijnders BJ, Peetermans WE, Verwaest C, Wilmer A, Van Wijngaerden E. Watchful waiting versus immediate catheter removal in ICU patients with suspected catheter-related infection: a randomized trial. Intensive Care Med. juin 2004;30(6):1073‑80.

53.

Rumbak MJ, Newton M, Truncale T, Schwartz SW, Adams JW, Hazard PB. A prospective, randomized, study comparing early percutaneous dilational tracheotomy to prolonged translaryngeal intubation (delayed tracheotomy) in critically ill medical patients. Crit Care Med. août 2004;32(8):1689‑94.

54.

Semler MW, Weavind L, Hooper MH, Rice TW, Gowda SS, Nadas A, et al. An Electronic Tool for the Evaluation and Treatment of Sepsis in the ICU: A Randomized Controlled Trial. Crit Care Med. août 2015;43(8):1595‑602.

55.

Tidswell M, Tillis W, Larosa SP, Lynn M, Wittek AE, Kao R, et al. Phase 2 trial of eritoran tetrasodium (E5564), a toll-like receptor 4 antagonist, in patients with severe sepsis. Crit Care Med. janv 2010;38(1):72‑83.

56.

Trzeciak S, Glaspey LJ, Dellinger RP, Durflinger P, Anderson K, Dezfulian C, et al. Randomized controlled trial of inhaled nitric oxide for the treatment of microcirculatory dysfunction in patients with sepsis*. Crit Care Med. déc 2014;42(12):2482‑92.

57.

Valette X, Desmeulles I, Savary B, Masson R, Seguin A, Sauneuf B, et al. Sodium Bicarbonate Versus Sodium Chloride for Preventing Contrast-Associated Acute Kidney Injury in Critically Ill Patients: A Randomized Controlled Trial. Crit Care Med. avr 2017;45(4):637‑44.

58.

Van den Berghe G, Wilmer A, Hermans G, Meersseman W, Wouters PJ, Milants I, et al. Intensive insulin therapy in the medical ICU. N Engl J Med. 2 févr 2006;354(5):449‑61.

59.

Ventura AMC, Shieh HH, Bousso A, Góes PF, de Cássia F O Fernandes I, de Souza DC, et al. Double-Blind Prospective Randomized Controlled Trial of Dopamine Versus Epinephrine as First-Line Vasoactive Drugs in Pediatric Septic Shock. Crit Care Med. nov 2015;43(11):2292‑302.

60.

Vet NJ, de Wildt SN, Verlaat CWM, Knibbe CAJ, Mooij MG, van Woensel JBM, et al. A randomized controlled trial of daily sedation interruption in critically ill children. Intensive Care Med. févr 2016;42(2):233‑44.

61.

Vignon P, Dequin P-F, Renault A, Mathonnet A, Paleiron N, Imbert A, et al. Intermittent pneumatic compression to prevent venous thromboembolism in patients with high risk of bleeding hospitalized in intensive care units: the CIREA1 randomized trial. Intensive Care Med. mai 2013;39(5):872‑80.

62.

Vlasselaers D, Milants I, Desmet L, Wouters PJ, Vanhorebeek I, van den Heuvel I, et al. Intensive insulin therapy for patients in paediatric intensive care: a prospective, randomised controlled study. Lancet. 14 févr 2009;373(9663):547‑56.
