# Supplementary material for: Methodological management of end-of-life decision data in intensive care studies: A systematic review of 178 randomized control trials published in seven major journals
Source: PLoS One. 2019 May 28;14(5):e0217134. doi: 10.1371/journal.pone.0217134 (PMC6538318; doi:10.1371/journal.pone.0217134)
Supplement: S2 Text — (DOCX) [file pone.0217134.s002.docx]

S2 Text. Codebook of variables collected for the systematic review

| **Name of the variable** | **details** |
| --- | --- |
| **id** | Id of the article in the database |
| **title** | Title of the article |
| **PMID** | PMID number in PubMed |
| **journal** | Name of the journal |
| **Year** | When the study take place |
| **Year_Pub** | Year of publication of the article |
| **type_essai** | Type of study |
| **intervention** | Type of intervention evaluated |
| **type_de_patient** | Type of ward (ICU, ER) |
| **survie** | Evaluation of mortality (y/n) |
| **mortalité** | Type of end point for mortality |
| **eligible** | Included in the systematic review |
| **end_point** | Primary end point of the study |
| **pays** | Country of the auteur |
| **aveugle** | Was the study blinded (y/n) |
| **centre** | One or more than one center |
| **nbre_de_centres** | Number of center |
| **inter** | Was the study international or not |
| **nbre_de_patients** | Number of patient included |
| **age_moyen** | Mean age of patients |
| **nbre_de_bras** | Number of arm |
| **nbre_patient_bras_ttt** | Number of patients in intervention arm |
| **nbre_patient_bras_control** | Number of patients in the control arm |
| **pathologie** | Type of pathology studied |
| **rea** | Type of ICU |
| **m_service_glob** | Global ICU mortality rate |
| **m_service_ttt** | Treatment arm ICU mortality rate |
| **m_service_ctrl** | Control arm ICU mortality rate |
| **m_hospi_glob** | Global HOSPITAL mortality rate |
| **m_hospi_ttt** | Treatment arm HOSPITAL mortality rate |
| **m_hospi_ctrl** | Control arm HOSPITAL mortality rate |
| **m_j28_glob** | Global DAY 28 mortality rate |
| **m_j28_ttt** | Treatment arm DAY 28 mortality rate |
| **m_j28_ctrl** | Control arm DAY 28 mortality rate |
| **m_60_glob** | Global DAY 60 mortality rate |
| **m_60_ttt** | Treatment arm DAY 60 mortality rate |
| **m_60_ctrl** | Control arm DAY 60 mortality rate |
| **m_90_glob** | Global DAY 90 mortality rate |
| **m_90_ttt** | Treatment arm DAY 90 mortality rate |
| **m_90_ctrl** | Control arm DAY 90 mortality rate |
| **m_180_glob** | Global DAY 180 mortality rate |
| **m_180_ttt** | Treatment arm DAY 180 mortality rate |
| **m_180_ctrl** | Control arm DAY 180 mortality rate |
| **m_360_glob** | Global DAY 360 mortality rate |
| **m_360_ttt** | Treatment arm DAY 360 mortality rate |
| **m_360_ctrl** | Control arm DAY 360 mortality rate |
| **periode_inclusion_début** | Beginning of inclusion period (dd/mm/yyyy) |
| **periode_inclusion_fin** | Ending of inclusion period (dd/mm/yyyy) |
| **inclusion_durée** | Inclusion length in month |
| **critere_pricipal** | Primary outcome |
| **critere_secondaire_1** | Secondary outcome number 1 |
| **critere_secondaire_2** | Secondary outcome number 2 |
| **critere_secondaire_3** | Secondary outcome number 3 |
| **critere_secondaire_4** | Secondary outcome number 4 |
| **critere_inclusion** | Patients inclusion criteria |
| **regression** | Use of a regression model (y/n) |
| **regression_modele** | If yes type of regression model |
| **itt** | Was the study analyzed in Intention To Treat principle (y/n) |
| **Type d'analyse statistique** | Type of analysis used for mortality (percentage, log rank, cox, other) |
| **lata_info** | Were information about End of Life decision management available in the full text (y/n) |
| **lata_obs** | Were End of Life decision observed in the full text or the protocol of the study (y/n) |
| **type_lata** | What type of ELD were observed (DNR, withholding, withdrawing, full life support, other) |
| **nbre_lata** | Global number of patients concerned by ELD in the study |
| **nbre_lata_ttt** | Number of patients concerned by ELD in the treatment group |
| **nbre_lata_ctrl** | Number of patients concerned by ELD in the control group |
| **methode ELD** | Method used to take into account ELD (non-inclusion, exclusion, considered as global mortality |
| **censure** | Were ELD data censored like survival data (y/n) |
| **censure_moment** | When were ELD data censored (time of decision, time of death, other) |
| **analyse_sensi** | Did author perform a sensibility analysis for the ELD data (y/n) |
| **flow_chart** | Was a flow chart available in publication (y/n) |
| **lata_flow_chart** | Did the flow chart contained information about ELD (y/n) |
| **inclus_RS** | Inclusion of the article in qualitative analysis (y/n) |
| **registre** | Was the study registered online (y/n) |
| **type_registre** | Type of register (Clinical trial.gov, other) |
| **registre_ple** | Was primary outcome of study the same that online |
| **sequence generation** | Was allocation generated randomly |
| **blinding of participant** | Was the study blinded to participant (y/n) |
| **blinding of outcome** | Was the study blinded to evaluation (y/n) |
| **other source of bias** |  |
