# Supplementary material for: Methodological management of end-of-life decision data in intensive care studies: A systematic review of 178 randomized control trials published in seven major journals
Source: PLoS One. 2019 May 28;14(5):e0217134. doi: 10.1371/journal.pone.0217134 (PMC6538318; doi:10.1371/journal.pone.0217134)
Supplement: S1 Fig — (DOCX) [file pone.0217134.s004.docx]

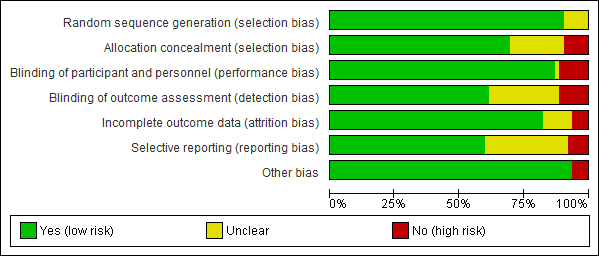


**Risk of bias graph: review authors' judgements about each risk of bias item presented as percentages across all included studies.**

**
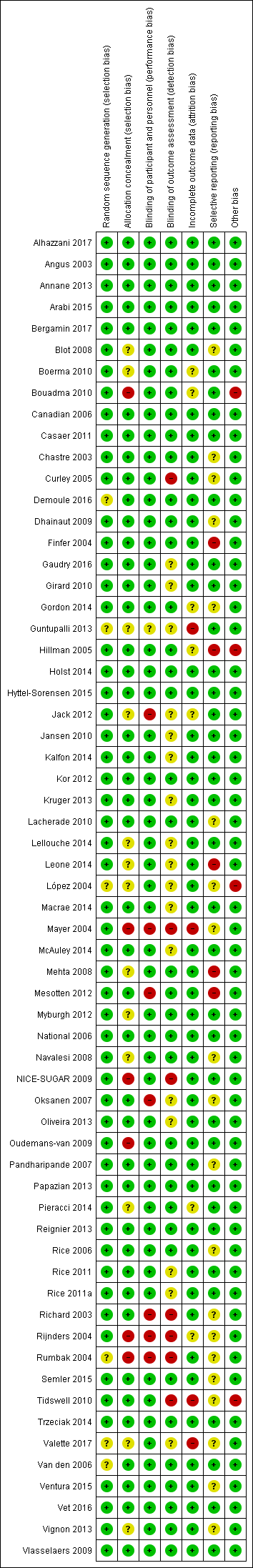
**

**Risk of bias summary: review authors' judgements about each risk of bias item for each included study.**
